# The role of performance pressure, loneliness and sense of belonging in predicting burnout symptoms in students in higher education

**DOI:** 10.1371/journal.pone.0267175

**Published:** 2022-12-21

**Authors:** Jolien M. Dopmeijer, Christine A. E. Schutgens, F. Rutger Kappe, Nikkie Gubbels, Tommy L. S. Visscher, Ellen M. M. Jongen, Rob H. L. M. Bovens, Jannet M. de Jonge, Arjan E. R. Bos, Reinout W. Wiers

**Affiliations:** 1 Trimbos-instituut, Netherlands Institute of Mental Health and Addiction, Utrecht, The Netherlands; 2 Department of Psychology, Addiction Development and Psychopathology (ADAPT-)lab, Centre for Urban Mental Health, University of Amsterdam, The Netherlands; 3 Teachers College, Windesheim University of Applied Sciences, Zwolle, The Netherlands; 4 Institute of Sport and Exercise Studies, HAN University of Applied Sciences, Nijmegen, The Netherlands; 5 Faculty of Psychology, Open University, Heerlen, The Netherlands; 6 Department of Education and Innovation, Study Success, Inholland University of Applied Sciences, Haarlem, The Netherlands; 7 Quality Assurance Department, Codarts University of the Arts, Rotterdam, The Netherlands; 8 Staff Office Education and Research, Hanze University of Applied Sciences, Groningen, The Netherlands; 9 Tranzo, Scientific Center for Care and Wellbeing, University of Tilburg, Tilburg, The Netherlands; 10 Faculty of Health, ACHIEVE, Centre of Applied Research, Amsterdam University of Applied Sciences, Amsterdam, the Netherlands; Universitat de Valencia, SPAIN

## Abstract

Student burnout is raising an increasing amount of concern. Burnout often leads to psychosocial problems and drop-out. In this study multiple regression analysis was used to examine the impact of performance pressure, loneliness, and sense of belonging on the underlying dimensions of burnout in 3,134 university students in the Netherlands. Results suggest that sense of belonging could be targeted as a way to enhance student wellbeing, in order to improve the ability to cope with the high demands in student life and the prevention of burnout.

## Introduction

For many students, their time at university is stressful, marked with many new experiences, challenges and life events [1]. Perhaps stronger than previously, student life is about meeting high expectations: getting good grades, building a resume and a good career start next to a rich social life [2]. Recent reports show an increase in perceived performance pressure among students leading to psychosocial problems, particularly burnout symptoms [2,3]. Accordingly, several studies have shown that experiencing burnout symptoms has become a common problem among students in higher education, with the reported prevalence ranging between 45 and 71% [1,4–6]. Burnout symptoms often lead to students feeling less engaged in their studies, poor academic performance, study delay and drop-out [1,7]. Over the past years, researchers have found that those students who report feelings of burnout are more likely to experience poor physical health and cardio-vascular disease [8], psychological distress such as depression and even suicidal ideation [4].

Burnout refers to a state of psychological exhaustion and is originally known as a work-related phenomenon defined as ‘a psychological syndrome as reaction to chronic interpersonal stressors at work’ [9]. This reaction consists of feeling exhausted, experiencing depersonalization, and feeling incompetent. Although the concept of burnout has originally been defined in the context of work-related stress, the traditional concept and scope have recently been broadened to include study-related problems [10]. Indeed, research on burnout symptoms among students in higher education is upcoming, showing that burnout symptoms among students seem even more prevalent than among the working population [5,6,11,12]. Burnout symptoms among students are similar to burnout symptoms among professionals, they refer to feelings of exhaustion (because of high study demands), having a cynical and detached attitude toward one’s study and experiencing a reduced sense of accomplishment as a student [10].

The development of burnout is explained by Job Demands-Resources model for Burnout and Engagement (JD-R Model). The model assumes that high demands lead to stress reactions and exhaustion, while having many resources (sources of energy), like rewards or social support, leads to higher productivity, which is a motivational process [13,14]. Burnout symptoms are a result of insufficient balance between experienced demands and resources [14]. While the JD-R model finds its origin in workplace settings, the model has also shown to be valid in the educational setting according to a study by Robins et al (2015) [15]. This study showed that mediation effects of study demands and (personal) resources (such as emotional stability and belonging) with psychological flexibility were found. The study found that the JD-R model contributed to the knowledge about burnout and engagement in university students, especially with regard to personal resources. The relationship between performance pressure and burnout symptoms is relevant to investigate. The increase of both perceived performance pressure and burnout symptoms makes it plausible that performance pressure could be a demand that contributes to the development of burnout symptoms among students and therefore could be a predictor of burnout symptoms.

Together with the increase in studies regarding burnout symptoms among students, there is a growing interest in loneliness among students. Loneliness can be defined as *‘the subjective experience of an unpleasant or unacceptable lack of (quality of) certain social relationships’* [16]. Several studies show that loneliness is a common problem among students and that loneliness and experienced study stress appear to explain depressive symptoms [17]. Another study shows that there also appears to be a relationship between loneliness and (lack of) study progress: lonely students experience more stress, are less successful in their final exams and are less involved in their studies [18]. An overview study shows loneliness to be related to cardiovascular diseases, the functioning of the immune system, premature death, fatigue, depression and anxiety [19]. Both loneliness and burnout are related to negative psychological health. The relationship between these concepts was investigated by Lin and Huang among a sample of Taiwanese university students and by Stoliker and Lafreniere among a sample of Canadian undergraduate students. These studies show that there’s a relationship between loneliness and burnout symptoms among students [1,20] and that loneliness is also a predictor of students’ engagement in their studies [1]. According to Ponzetti [21], students with feelings of loneliness may also be experiencing increased levels of social detachment, which is one of the dimensions of burnout, called *depersonalization* in the JD-R Model. Given the results of these previous studies, it is likely that loneliness plays a role in the development of burnout symptoms. It’s likely that the loneliness can be seen as a demand.

The other related concept to study resources is sense of belonging, which refers to *‘feelings of safety and comfort that arise from the idea of being part of a community*, *organization or institution’* [22]. The most important dimensions of sense of belonging are the feeling of being appreciated and the feeling of fitting within the group. Sense of belonging is a widely studied concept within the educational setting, primarily focused on the relationship with academic performance and motivation. It is seen as an important condition for learning, since learning is generally not considered as an individual, but social process that takes place through collaboration. Students with a higher sense of belonging are more motivated, more committed to their studies and have a greater intention to continue studying [23–26]. On a smaller scale, research has been done regarding the relationship between sense of belonging and mental health of students. A higher sense of belonging was found to be associated with fewer study problems and fewer depression symptoms [27], where another study shows that a lower sense of belonging explained the significantly higher degree of psychosocial problems experienced by children of immigrants, who were the first to go to university [28]. Although little research has been done regarding the relationship between sense of belonging and burnout symptoms, a Norwegian study shows a negative relationship between sense of belonging and one of the dimensions of burnout, *exhaustion* [29].

For a long time, the concepts of loneliness and sense of belonging were seen as two sides of the same coin, as a result of which they’ve rarely been investigated together. Although both concepts stem from the need for connectedness, they differ from each other. Loneliness arises from a discrepancy between the desired and the perceived connectedness with others, which depends on someone’s expectations of friendship or intimate relationships and what they deliver. Sense of belonging depends on the degree of being involved and valued on the one hand, and on the other having the feeling of fitting into a system or environment. Relationships are part of that, but not the only thing that makes someone feel fitting in [22]. Another difference is that sense of belonging is context specific and loneliness is not [22]. Because burnout is also context specific and the feeling of fitting into a school environment could be a resource that is more comprehensive than just the satisfaction about social relationships, unlike loneliness, sense of belonging is likely to be a resource and may be a specific and important protective factor for burnout symptoms.

It is clear that studies show that burnout symptoms have severe consequences for students, what makes it of great importance to gain insight into the factors that are related to the development of these symptoms and the key principles that can contribute to its prevention. Although several studies have shown that there are indications that performance pressure, loneliness and sense of belonging are predictive of burnout symptoms in general, little is known about what predicts burnout among students. The main aim of this study is to examine if there is a relationship between performance pressure, loneliness and sense of belonging on the one hand and the three dimensions of burnout (i.e., emotional exhaustion, depersonalization and reduced sense of personal accomplishment) on the other hand among students in higher education.

## Materials and methods

### Procedure

Cross-sectional data were used from the Study environment, Health and Study Success online survey, carried out between December 2017 and March 2018 in a large university of applied sciences in the Netherlands. Fulltime students were invited by email (*N* = 18,163). They were informed upfront about the objective and procedure of the study and participated voluntarily. The consent of the participants was obtained by virtue of survey completion.

### Measures

#### Outcomes

*Burnout* was assessed with the validated Dutch version of the Maslach Burnout Inventory General Survey (MBI-GS), the Utrecht Burnout Scale General Version (UBOS-A) [30], that was adapted for use in our student sample. For instance, the item *“I feel mentally drained from my job”* was rephrased in *“I feel mentally drained from my studies”*. The UBOS-A questionnaire consists of 15 items that constitute 3 subscales (or dimensions) that together measure burnout symptoms: Emotional Exhaustion (EE; Cronbach’s alpha .87 in the present study), Depersonalization (DP; Cronbach’s alpha .87 in the present study) and a reduced sense of Personal Accomplishment (PA; Cronbach’s alpha.73 in the present study, obtained by removal of the item *“I just want to do my studies and not be bothered”*, which made the alpha considerably higher, i.e., from .60 to .73). All 14 items are scored on a 7-point Likert scale from 1 (*never*) to 7 (*always*). The average scale scores of the three UBOS-A scales are calculated by adding up the scores and dividing by the number of items in the scale of question. High scores on Emotional Exhaustion (EE, mean score ≥2.39) and Depersonalization (DP, mean score ≥ 2.25) and low scores on Personal Accomplishment (PA, mean score ≤.49) are indicative for burnout [7].

#### Predictors

*Performance pressure* was assessed with the question: *‘To what extent do you feel that you have to perform or achieve*?*’* The item was scored on a 5-point Likert scale from 1 (never) to 5 (very often). The higher the score, the more performance pressure was experienced. This scale yielded an average of 2.85 (*SD* = .88).

*Loneliness* was assessed with the De Jong-Gierveld Loneliness Scale with a Cronbach’s alpha of .93 in the present study [16]. This scale consists of the emotional loneliness subscale (5 items) and the social loneliness subscale (6 items) which together measure loneliness. An example from the emotional loneliness subscale is: *“I experience a general sense of emptiness”* and an example of the social loneliness subscale is: *“There are plenty of people I can rely on when I have problems”*. All items were scored from 1 (Yes! Totally agree) to 5 (No! Totally disagree). First, the emotional loneliness score is computed by counting all neutral and positive answers on the negatively formulated items and second, the social loneliness score is computed by counting all neutral and negative answers on positively formulated items. The total loneliness score (0 to 11) is computed by taken the sum of the emotional loneliness score and the social loneliness score. This scale yielded an average of 23.41 (*SD* = 8.29) and a Cronbach’s alpha of .93 in the present study.

*Sense of belonging* was assessed by the Sense of Belonging questionnaire [25], consisting of 6 items, for example: “*I feel at home at this university of applied sciences*.*”* All items are rated on a 5-point Likert scale from 1 (not true at all) to 5 (completely true). An average score of the items was calculated for each participant. The higher the total score, the more the respondent experienced a sense of belonging. This scale yielded an average of 3.81 (*SD* = .60) and a Cronbach’s alpha of .77 in the present study.

#### Covariates

Covariates *age*, *year of study* and *gender* were obtained from data of the Student Administration of the university. *Age* was expressed in years, and *gender* was coded dichotomously (1 = *female*, 2 = *male*). The students’ *year of study* was either *1*^*st*^ *year* (= 1), *2*^*nd*^ *year* (= 2), *3*^*rd*^ *year* (= 3), *4*^*th*^ *year* (= 4) or *higher than 4*^*th*^ *year* (= 5). *Living situation* was assessed as a self-reported item and was also coded dichotomously (1 = *living independently*, 2 = *living with parents*).

### Statistical analysis

Sociodemographic characteristics and descriptive values for all variables were calculated using IBM SPSS Statistics, version 23 [31]. Multiple regression analysis using Muthén and Muthén’s (1998–2011) software MPLUS version 8.0 [32], was used to test the predictive role of performance pressure, sense of belonging and loneliness in burnout symptoms. All three predictors in the model, i.e. emotional exhaustion, depersonalization and (lack of) personal accomplishment, which together predict burnout symptoms, were analyzed together to control for their shared variance in burnout symptoms. Main effects were analyzed while controlling for demographic covariates (age, gender, living situation and year of study).

Maximum likelihood with robust standard error (MLR) was used as estimation method in order to control for possible non-normality’s of the data. Preliminary analyses were performed and it was ensured that there were no violations of the assumption of normality, linearity, multicollinearity and homoscedasticity.

## Results

A total of 3,141 students completed the “Study Environment, Health and Study Success” survey (response rate 17.3%), 60% of whom were female students (*N* = 1886) and 40% of whom were male students (*N* = 1255) (Table 1). Their average age was 21.8 years old (*SD* = 3.45). Of all students 70.6% were living with their parents and 29.4% were living independently. A total of 28.1% of the participants were freshman (first year) and 71.9% were students in later years. Almost two-third (68.9%) of the participants said that they experienced performance pressure often to very often. Almost half of the participants experienced loneliness (43.3%) and the average score for sense of belonging was 3.81 (*SD* = .60).

**Table 1:** Sample characteristics (*N* = 3,141).

Variable	% Participants (or mean with SD)
**Gender**	
Male	40.0
Female	60.0
**Age** (*mean*, *SD*)	21.8 (3.45)
**Ethnicity**	
Non-Dutch	11.6
Dutch	88.4
**Living situation**	
Living with parents	70.9
Living independently	29.4
**Year of study**	
1st	28.1
2nd	21.3
3rd	21.8
4th	18.4
Higher than 4th	10.4
**Burnout**	
Emotional Exhaustion subscale (*high-very high*)	60.2
Depersonalization subscale (*high-very high*)	34.4
Personal accomplishment scale (*very low-low*)	53.2
Burned out	44.2
**Performance pressure**	
Often or very often	68.9
**Loneliness**	
Not	56.7
Moderate to very severe	43.3
**Sense of belonging** (*mean*, *SD*)	3.81 (0.60)

Results of the correlations analyses are shown in Table 2. This table shows that although some variables appeared to be correlated, no high intercorrelations occurred among variables of our multiple regression model. This shows that there were no violations of the assumption of normality, linearity, multicollinearity and homoscedasticity.

**Table 2:** Correlations (N = 3,141).

	**Emotional exhaustion**	**Depersonalization**	**Personal accomplishment**	**Loneliness**	**Performance pressure**	**Sense of belonging**
**Emotional exhaustion**	1					-
**Depersonalization**	.57**	1				
**Personal accomplishment**	-.30**	-.45**	1			
**Loneliness**	.32**	.27**	-.28**	1		
**Performance pressure**	.37**	.15**	-.09**	.15**	1	
**Sense of belonging**	-.35**	-.55**	.39**	-.29**	-.07**	1

** *p* < .01.

Results of the multiple regression analysis are shown in Table 3. Performance pressure, loneliness and sense of belonging were significantly associated with the three dimensions of burnout: emotional exhaustion, depersonalization, and personal accomplishment, adjusting for gender, age, living situation and year of study. All these associations reached statistical significance, except one: the association between performance pressure and personal accomplishment.

**Table 3:** Emotional exhaustion, depersonalization and personal accomplishment explained by multiple regression analysis (N = 3,141).

		Emotional Exhaustion			Depersonalization			Personal Accomplishment		
	Variable	Standardized β	Standard Error	p-value	Standardized β	Standard Error	p-value	Standardized β	Standard Error	p-value
**Predictors**	Performance pressure	.29	.02	.000	.10	.02	.000	.00	.02	.817
	Loneliness	.19	.02	.000	.12	.02	.000	-.15	.02	.000
	Sense of belonging	-.28	.02	.000	-.47	.02	.000	.23	.02	.000
**Covariates**	Gender	-.11	.02	.000	.04	.02	.006	.08	.02	.000
	Age	-.07	.02	.000	-.05	.02	.003	.02	.02	.338
	Living situation	.02	.02	.242	-.02	.02	.299	-.02	.02	.445
	Year of study	.07	.02	.000	.21	.02	.000	-.08	.02	.000
		**R**^**2**^ **= .288**			**R**^**2**^ **= .365**			**R**^**2**^ **= .107**		

### Emotional exhaustion

Table 2 shows that performance pressure, loneliness and sense of belonging explained 28.8% of the variance in emotional exhaustion, while adjusting for gender, age, living situation and year of study. Performance pressure, loneliness and sense of belonging were all significantly predicting emotional exhaustion independently from each other (all p-values < .01). Background variables that contributed significantly (*p* < .05) to the prediction of emotional exhaustion were gender, age, and year of study. Exhaustion was more present among younger, female students and students that are longer on campus. Living situation did not explain emotional exhaustion significantly.

### Depersonalization

Regarding depersonalization, Table 2 shows that performance pressure, loneliness and sense of belonging explained 36.5% of its variance, while adjusting for gender, age, living situation and year of study. Performance pressure, loneliness and sense of belonging were all significantly predicting depersonalization independently from each other (all p-values for beta < .01). Comparing the standardized regression coefficients (beta’s) shows that sense of belonging (*β* = -.47) appeared to be the strongest predictor of depersonalization compared to performance pressure (*β* = .10) and loneliness (*β* = .12). Background variables that contributed significantly (*p* < .05) to the prediction of depersonalization (*p* < .05) were age, gender and year of study. Living situation did not contribute significantly. Depersonalization was more present among students that were longer on campus.

### Personal accomplishment

Regarding (the lack of) Personal Accomplishment, Table 2 shows that performance pressure, loneliness and sense of belonging explained 10.7% of its variance, while adjusting for gender, age, living situation and year of study. Loneliness and sense of belonging were both significantly predicting personal accomplishment independently from each other (all p-values for beta < .01). Performance pressure did not significantly predict personal accomplishment.

Background variables that contributed significantly (*p* < .05) to the prediction of personal accomplishment (*p* < .05) were gender and year of study. A sense of reduced personal accomplishment was more present among male students and students that were longer on campus. Age and living situation did not contribute significantly.

## Discussion and conclusions

### Key results

Our results show that performance pressure, loneliness and sense of belonging are significant predictors of burnout symptoms among students, especially of the facets relating to emotional exhaustion and depersonalization. Sense of belonging appeared to be the strongest predictor of all burnout symptoms. Our results are in line with the JD-R model, explaining that burnout symptoms are caused by an imbalance between demands and resources, where high demands lead to emotional exhaustion and low resources ensure depersonalization [14].

A study by Maslach, Schaufeli & Leiter [9] shows that work pressure plays an important role in the development of burnout symptoms. Jacobs & Dodd [33] show that this also applies to performance pressure among students, just like in the present study. Both studies show that performance pressure is most strongly related to the development of emotional exhaustion and less to depersonalization and personal accomplishment. It is likely that students who experience performance pressure work harder. Eventually, for most students it leads to symptoms of exhaustion, which affects their functioning.

The present study shows that exhaustion and depersonalization were more present among students that are longer on campus and that exhaustion was more present among female students, where depersonalization and a sense of reduced personal accomplishment were more present among male students. This is in line with previous studies according to a meta-analysis by Purvana and Muros [34] that states that women are more likely to report emotional exhaustion and men are more likely to report depersonalization, but these gender differences lay in the close-to-zero effect size range. Our findings regarding the higher presence of exhaustion and depersonalization among students that are longer on campus support findings of a study by Salmela-Aro & Read [35]. They state that burnout symptoms increase along with the numbers of years of study. We believe that further study into the issue of gender, age and years on campus differences is necessary.

The appeared predictive value of sense of belonging confirms the important role of the university context in the development of burnout symptoms. Hence, sense of belonging is mostly about feeling at home at university. Students’ sense of belonging increases as they feel a stronger connection with their university and the people there [22]. Thus, the feeling of not belonging within the university context increases the risk of emotional exhaustion and depersonalization: symptoms of burnout. Loneliness appeared to be a weaker predictor of burnout symptoms than sense of belonging was. Loneliness can arise without being related to the study context. This could explain why students can report low levels of sense of belonging and high level of burnout symptoms in their studies, while not reporting loneliness.

In addition to previous findings of Bakker, Demerouti, & Euwema [36], the present study also shows a relationship between resources and emotional exhaustion. Sense of belonging and social support appear to be important resources here that provide energy and reduce the chance of getting emotional exhausted. Possibly, when students feel connected to the study program and at the same time feel supported during this program, study tasks cost less energy and exhaustion, therefore burnout symptoms, are prevented.

### Limitations

The measurement of performance pressure is debatable. Because performance pressure among students is a fairly new concept, no reliable and valid questionnaire was available. This also might have led to limitations regarding the estimation of perceived performance pressure in students. However, our findings were in line with previous studies that are firmly rooted in the theory of the JD-R model.

Furthermore, because cross-sectional data were used, causal interpretations require caution. Students’ experiences are subject to change over time. As a result, it is unknown how burnout symptoms develop over time. Longitudinal data are needed to confirm that students’ experiences predict burnout and that changes in students’ experiences predict changes in burnout levels. Future studies should also focus on the occurrence and consequences of burnout and performance pressure among a broader sample of several universities, based on a new definition of both burnout and performance pressure specifically for students.

Finally, the results of the present study are based on a Dutch sample. An advantage of the present study is the large sample that was found to be representative for the total population of the university where the study was conducted in terms of the mean age and distribution of students’ gender, faculties and year of study, which contributes to validated conclusions. The extent to which burnout symptoms exist may differ between universities, however, there’s no reason to assume that the associations found in the present study will differ greatly between universities.

### Implications and conclusions

This study highlights the importance of including sense of belonging, loneliness and performance pressure in future studies of student burnout. There’s a need for a better understanding of what underlies performance pressure, loneliness and burnout and how we can adequately address these factors. There are indications that performance pressure, loneliness and burnout symptoms are not only rooted within the educational environment, but also in a broader societal context [2,3]. In order to address these problems efficiently, it is important to examine the origin of performance pressure, loneliness and burnout symptoms and the factors that may be of influence within the educational setting thoroughly.

Our findings show that performance pressure and loneliness as demands, and sense of belonging as a resource predict burnout symptoms in students. Performance pressure, as one of the greatest concerns of this time regarding student wellbeing, is strongly associated with mainly emotional exhaustion, but also with depersonalization. Performance pressure can therefore be seen as an important demand during student life. There is a need to examine the association between performance pressure, loneliness and burnout symptoms on the one hand and academic performance on the other hand in more extended longitudinal studies, with a starting point in the first year when students have no academic experience yet.

Our findings increase the understanding of burnout development among students from a more contextual perspective. They show the importance of the study environment as protective factor for the development of burnout symptoms. A lack of sense of belonging showed the highest, significantly increased risk for all three dimensions of burnout. Therefore, sense of belonging appears to be essential as a resource that leads to the prevention of burnout symptoms. This is not surprising, since the association between sense of belonging and positive mental health is well demonstrated [28]. Several studies suggest that sense of belonging is a significant predictor of both academic success and student mental health [37–40]. Students who report a high sense of belonging adjust better to university life [39] and report lower levels of depression and loneliness [38]. Bakker, Hakanen, Demerouti, & Xanthopoulou [41] found that resources like social support and appreciation enhance the sense of belonging and mitigate the negative effect of high performance pressure. We therefore suggest to make sense of belonging a priority in the enhancement of student wellbeing, as this resource could improve the ability of coping with the high demands in student life.

What happens to student wellbeing and academic performance if you enhance resources like social support and a supportive study environment? With a better understanding of both the course and consequences of burnout and possible other psychosocial problems and protective factors that make prevention of these problems possible, we will be able to promote the wellbeing and student success of students in higher education.

## Supporting information

S1 File (SAV)
